# Sticking to rough surfaces using functionally graded bio-inspired microfibres

**DOI:** 10.1098/rsos.161105

**Published:** 2017-06-07

**Authors:** Serdar Gorumlu, Burak Aksak

**Affiliations:** Department of Mechanical Engineering, Texas Tech University, Lubbock, TX 79409-1021, USA

**Keywords:** roughness, adhesion, bioinspired adhesives, gecko, pull-off, functionally graded microfibres

## Abstract

Synthetic fibrillar adhesives inspired by nature, most commonly by the gecko lizard, have been shown to strongly and repeatedly attach to smooth surfaces. These adhesives, mostly of monolithic construction, perform on par with their natural analogues on smooth surfaces but exhibit far inferior adhesive performance on rough surfaces. In this paper, we report on the adhesive performance of functionally graded microfibrillar adhesives based on a microfibre with a divergent end and a thin soft distal layer on rough surfaces. Monolithic and functionally graded fibre arrays were fabricated from polyurethanes and their adhesive performance on surfaces of varying roughness were quantified from force–distance data obtained using a custom adhesion measurement system. Average pull-off stress declined significantly with increasing roughness for the monolithic fibre array, dropping from 77 kPa on the smoothest (54 nm RMS roughness) to 19 kPa on the roughest (408 nm RMS roughness) testing surface. In comparison, pull-off stresses of 81 kPa and 63 kPa were obtained on the same respective smooth and rough surfaces with a functionally graded fibre array, which represents a more than threefold increase in adhesion to the roughest adhering surface. These results show that functionally graded fibrillar adhesives perform similar on all the testing surfaces unlike monolithic arrays and show potential as repeatable and reusable rough surface adhesives.

## 1. Introduction

Geckos have the ability to cling to both rough and smooth surfaces robustly as evidenced by the wide variety of the surfaces that they climb on in their natural habitat. The hierarchical fibrillar structure on its toe pads comprising lamellae, setae and spatulae is believed to equip the gecko with the ability to adapt to variation in surface roughness and generate adequate adherence to carry its body mass. For rough surfaces, the first necessary condition for overall adhesion is that setae can conform locally, and stick to, the adhering surface. This local roughness adaptation exists because the terminal end of a seta is decorated with a densely packed layer of fibres, called spatulae, that are sub-micrometre in diameter and several micrometres tall [1]. The spatula fibre array forms a layer analogous to a material with a relatively large effective compliance compared with the material fibres are made of [2]. Once adhesion at the setae level is satisfied, roughness on the global scale (i.e. a length scale associated with the size of the footpad) determines how the adhesive contribution from each seta in contact will add up to the overall adherence of the gecko [3,4]. Measurements carried out by Huber *et al*. [5] with individual seta demonstrates the ability of gecko to cope with local roughness. When they measured the pull-off force of single seta from rough surfaces with root mean square (RMS) roughness ranging from 20 nm to 1.1 μm, a minimum pull-off force of 10 nN was obtained for adhering surfaces of RMS roughness 100–300 nm. This represents a drop of only approximately 33% compared with 15 nN pull-off force obtained for the 20 nm RMS roughness. Interestingly, the pull-off force was observed to increase monotonically with increasing RMS roughness following this minimum. They obtained similar results from experiments performed with a living Tokay gecko. A qualitative analysis based on the onset of sliding of the live gecko on surfaces of varying roughness on a rotating platform showed that the geckos started sliding on surfaces of 90 nm RMS roughness at 135° slope. On the other hand, the gecko could stay attached briefly to the surface with 238 nm RMS roughness at 180° rotation (i.e. upside down position). The gecko was able to stay attached to the surface with 3 nm RMS roughness for 5 min. Pugno & Lepore [6] studied the adherence of Tokay geckos in a similar fashion where they measured the time of adhesion to surfaces of varying roughness and used the time of adhesion as a relative measure of adherence. They concluded that geckos were able to stay attached to the surface with 618 nm RMS roughness the longest, which is slightly different than the findings presented in [5]. While these studies use different methods to characterize the performance of the gecko on rough surfaces, their findings using rough surfaces of sub-micrometre to several micrometres RMS roughness suggest that the gecko seta is very adept at mitigating the effects of local roughness on adhesion.

On the synthetic side, focus has largely been on characterization of adhesion to smooth surfaces. Measured pull-off stresses obtained with gecko-inspired synthetic fibrillar adhesives, which include but are not limited to carbon nanotubes [7–9], mushroom-like microfibres [10–15] and hierarchical micro/nano fibres [16–18], have matched and in some instances surpassed adhesion strengths recorded for gecko footpads on smooth surfaces. These synthetic adhesives have either never been tested on rougher surfaces or performed significantly inferior to their biological counterparts on rough surfaces. A recent study in rough surface adhesion using micropillars generated approximately 0.1 N cm^−2^ (1 kPa) adhesion strength to a surface with peak-to-valley roughness of just 0.72 μm [19], 2 orders of magnitude lower than what the gecko can achieve. Similarly, mushroom-like PDMS (polydimethylsiloxane) pillars of 10 μm diameter and 20 μm height performed an order of magnitude worse for surfaces with 373 nm and higher RMS roughness compared with their adhesion to a smooth 2 nm RMS roughness surface, the pull-off stress dropping from approximately 100 kPa on the smooth to less than 10 kPa on the surfaces of 373–618 nm RMS roughness [20]. Active materials and methods such as shape memory polymers [21,22] and electrostatic effect [23] have been used successfully for enhancing the adhesion of fibrillar/post-like structures to both smooth and rough surfaces. It is, however, desirable to use a passive adhesive because the requirement for active control and external power limit the applications for such active methods. Note that the majority of the fibrillar adhesives to date have been of monolithic construction from materials with elastic moduli ranging from 10^6^–10^12^ Pa, and even at 10^6^ Pa, they lack the necessary compliance for local roughness adaptation.

As demonstrated by Yao & Gao [24], materials with elastic modulus gradient can be used to generate flaw-insensitive adhesion by limiting the interfacial separation to below a critical value such that the interfacial stress is equal to the theoretical adhesion strength *σ*_o_ at pull-off independent of the size of the flaw at the interface. Another method to obtain flaw-tolerant adhesion is to decrease the contact size such as observed in many biological attachment systems. For sufficiently small contacts, the interface is guaranteed to be under uniform stress of *σ*_o_ during separation, leading to the largest possible adhesion stress [25,26]. Thus, microfibrillar adhesives employing functionally graded materials combine these two methods and should lead to strong and flaw-tolerant adhesion, particularly on smooth surfaces.

The other advantage of such an adhesive system lies in its ability to locally adapt to roughness without compromising the attainable fibre fill factor. For a physical explanation, we use Persson's model for adhesion of elastic solids to rough surfaces and apply it to a single fibre [27]. Per this model, one can calculate an effective work of adhesion *w*_e_ to replace the thermodynamic work of adhesion *w* in a given pull-off force calculation. It is estimated using the energy balance between adhesion energy and the elastic energy stored to attain complete contact as

1.1 $$w_{e}=w\left[1+\pi \int_{q_{a}}^{q_{1}}\text{d}qq^{3}C(q)-\frac{\pi E}{2(1-\upsilon^{2})w}\int_{q_{a}}^{q_{1}}\text{d}qq^{2}C(q)\right].$$

Here, *q* is the wavevector, *q_a_* = 2π/*a_d_* is the magnitude of the wavevector corresponding to the contact size (fibre tip diameter in this case) and *q*_1_ is that corresponding to the interatomic distance, and *E* and *ν* are the elastic modulus and the Poisson's ratio of the soft material, respectively, if the adhering surface is rigid. The first two terms on the right-hand side of the equation represent the non-dimensional adhesion energy and the third term represents the non-dimensional elastic-stored energy. One can show using this equation that the effective work of adhesion will be higher for lower *E* because for a given rough surface less elastic energy will be stored for conformal contact. Indeed, for sufficiently soft materials, it is possible to obtain *w*_e_ > *w* because the gain in adhesion energy due to increased contact area with roughness can be much larger than the adverse effect of the increase in elastic-stored energy [27,28]. Then, why not use very soft materials for fibre construction? The problem lies with the fill factor: fibres made of softer materials will have to be spaced sufficiently apart to prevent them from clumping, which drastically decreases the fill factor [26]. With adhesives comprising monolithic soft fibres, gains in adhesion due to enhanced compliance will be negated due to low fibre fill factors. Functionally graded microfibres with soft tips and stiffer bases can achieve both good adhesion and high fill factors. This approach ensures that the local compliance is governed by the elastic modulus of the tip, whereas the fibre fill factor is determined by the base fibre elastic modulus. We propose that functionally graded microfibre adhesives where individual fibres use a heterogeneous elastic modulus can enhance adhesion by improving local roughness adaptation without adversely affecting the fibre fill factor.

The functionally graded fibres proposed in this work use the simplest form of functional grading, a step change in the elastic modulus: a mushroom-like base fibre and a thin soft distal layer. The mushroom-like geometry is selected due to its ability to uniformly distribute applied load at its interface [29,30], and the soft distal layer for improving local compliance. The strategy is to reduce the elastic-stored energy necessary to conform to the surface within the footprint of the fibre tip without adversely affecting the uniformity of the stress distribution. As suggested by Persson & Tosatti [27], this should lead to higher average adhesion stress for a given contact area. However, the thickness of the distal layer in relation of the tip diameter and surface roughness is critical and its effect will be shown experimentally and discussed further in the discussion section. In §2, a description of the fibre fabrication, details regarding dimensional characterization and adhesion measurements are given. Experimental results in §3 are followed by discussions of the results and their implications in §4. Conclusions of the study are given in §5.

## 2. Materials and methods

### 2.1. Fabrication of the functionally graded microfibres

**Figure 1.**
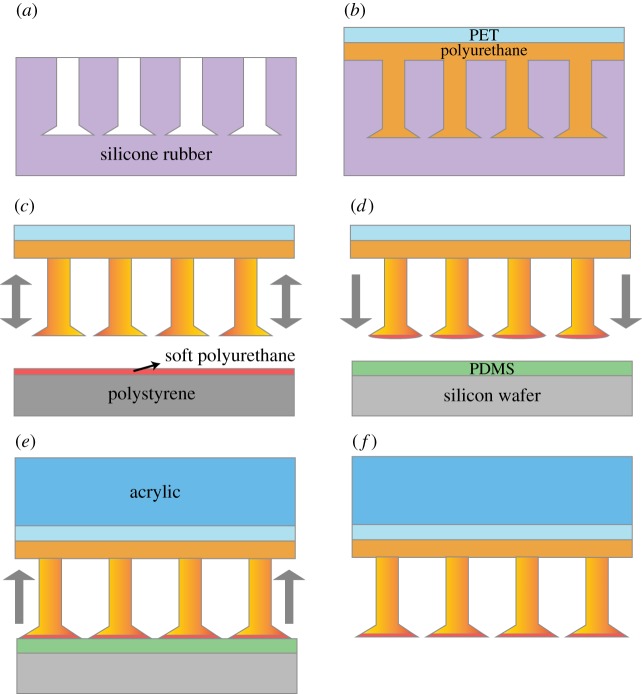
Schematics of the fabrication steps for functionally graded fibre arrays. (*a*) A shape complementary mushroom-like fibre mould is fabricated using casting. (*b*) Shape complementary mould is cast with polyurethane with a PET backing. (*c*) Fibre array is dipped into an uncross-linked thin layer of softer polyurethane. (*d*) The dipped sample is placed on a smooth PDMS layer. (*e*) An acrylic backing is glued to the fibre array using clear polyurethane. (*f*) The fibre array is peeled off from the PDMS layer.

**Figure 2.**
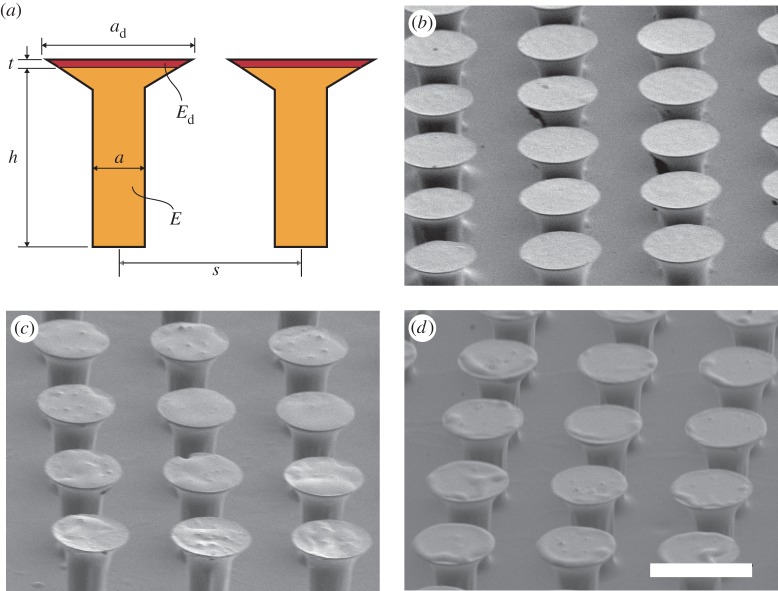
(*a*) Illustration of the relevant dimensions of a functionally graded microfibre. SEM images for (*b*) monolithic, (*c*) FG-4 µm and (*d*) FG-7 µm microfibre samples. Note that the thin soft layer is visible in (*c*) and (*d*) for the functionally graded fibre arrays. Scalebar, 100 µm.

The initial step in the fabrication of the functionally graded microfibres relies on the generation of a master template of mushroom-like fibres. Well-established methods detailed elsewhere were used to fabricate the master template and readers are referred to Gorumlu & Aksak [31] and Murphy *et al.* [13] for a detailed description. Additionally, the fabrication of the functionally graded fibres is based on a technique demonstrated by Bae *et al*. [32] where they used composite fibres to improve adhesion to skin. The master template of mushroom-like fibre array was first cast with silicone rubber (Moldmax 27T, Smooth On). Silicone rubber was cured at ambient temperature for 24 h and was peeled from the master template, forming the shape complementary mushroom-like microfibre mould (figure 1*a*). This mould was then cast with polyurethane of elastic modulus *E *= 8.89 MPa (M-3180, BJB Enterprises) and let cure for 24 h at ambient temperature with a 1 mm thick PET backing (figure 1*b*). Upon curing, fibres were peeled from the complementary mould to obtain the base fibres. Note that this step generated the monolithic mushroom-like fibre array. In a separate fabrication step, an uncross-linked polyurethane of elastic modulus *E*_d_ = 172 kPa (Vytaflex 10, Smooth On)) was spun onto a polystyrene Petri dish (100 × 15 mm, Fischer Scientific) at spin speeds of 6000 r.p.m. and 10 000 r.p.m. both for 2 min to obtain two different thicknesses of the liquid polyurethane layer. Monolithic mushroom-like fibres were then placed fibres facing down onto the thin layer and removed immediately retaining some of the liquid polymer on their tips (figure 1*c*). They were then placed onto a PDMS (1 : 10 weight ratio, Sylgard 184, Dow Corning) coated silicon wafer (spun coated at 4000 r.p.m. for 2 min and cured for 24 h at 70°C on a hotplate) to flatten the liquid polyurethane (figure 1*d*). As shown in figure 1*e*, after a curing step of 24 h at room temperature, a 1/8 inch thick 2.5 × 2.5 mm acrylic piece was glued onto the PET backing using a clear polyurethane (Crystal Clear 200, Smooth On). Clear polyurethane was let cure for 24 h at room temperature. The fibres were then peeled off the PDMS coated substrate with the acrylic backing (figure 1*f*). Note that the use of PDMS as the tip-forming substrate is critical in allowing the soft polyurethane to bond with the tip and come off the substrate without tearing off. Figure 2*a* illustrates the relevant dimensions of the intended fibre architecture.

Two functionally graded fibre arrays employing different distal layer thicknesses (FG-4 µm and FG-7 µm) and a monolithic fibre array were fabricated using the described fabrication method. The scanning electron microscope (SEM) images of the fabricated microfibres are shown in figure 2. The dimensions of the fibres were measured using optical profilometry (ContourGT-K, Bruker, USA). Since the functionally graded microfibres were fabricated from the monolithic microfibre array by adding a soft distal layer to individual fibres, the thickness of this layer was estimated from the height difference between the individual fibres in the functionally graded and the monolithic arrays. Table 1 includes the dimensions and the materials properties of the fibre arrays. The measurement uncertainty is 0.4 µm. Fibre tip diameter and height mean values and standard deviations are calculated from measurements of ten randomly selected fibres (see figure 2*a* for the description of the variables in the table).

**Table 1.** Material properties and dimensions of the fabricated fibre arrays.

sample	*E* (MPa)	*E*_d_ (MPa)	*a* (µm)	*a*_d_ (µm)	*h* (µm)	*t* (µm)	*s* (µm)
monolithic	8.89	—	40	70.6 ± 1.3	85.3 ± 0.1	—	120
FG-4 µm	8.89	0.172	40	71.9 ± 3.0	88.7 ± 0.8	3.4 ± 0.8	120
FG-7 µm	8.89	0.172	40	82.8 ± 3.3	91.7 ± 0.8	6.4 ± 0.8	120

### 2.2. Rough surface preparation and characterization

The effect of roughness on the adherence of monolithic and functionally graded fibre arrays was characterized from adhesion measurements on clear acrylic surfaces with five different roughness profiles. These surfaces were generated manually using sandpaper (P2000, Struers, USA) and cleaned using isopropyl alcohol, de-ionized water and clean dry air. The resulting surfaces had RMS roughness values ranging from 54 to 408 nm. Roughness of the testing surfaces was characterized using topography images obtained from a 1 × 1 mm scan area with the optical profilometer and for a smaller scan area of 60 × 60 µm with an atomic force microscope (AFM) (Dimension Icon, Bruker). The RMS roughness of the rough adhering surfaces is listed in table 2. The RMS roughness values obtained with the AFM are slightly lower than that obtained with the optical profilometer, as expected [33]. The surfaces are labelled with their RMS roughness values obtained from the optical profilometer for reporting purposes in the results section. The power spectral density plots of the rough adhering surfaces are included in electronic supplementary material, figure S1.

**Table 2.** The RMS roughness values of the experimental adhering surfaces.

	RMS roughmess (nm)	
surface	60 × 60 µm scan (atomic force microscope)	1 × 1 mm scan (optical profilometer)
54 nm RMS	39	54
239 nm RMS	234	239
300 nm RMS	255	300
408 nm RMS	348	408

### 2.3. Pull-off experiments

A custom adhesion measurement system was used to perform adhesion experiments between fibre arrays and rough surfaces. In short, the system consists of a precision linear stage (MFA-CC, Newport Corp.), a manual xy-stage (9067-XY, Newport Corp.), a 500-gr load cell (GSO-500, Transducer Techniques), an inverted microscope (Nikon Eclipse MA-100, Nikon) and a goniometer (M-GON-40-L, Newport Corp.). The system is autonomously operated using a LabVIEW (National Instruments, USA)-based custom software that controls preload, contact time, approach and retract speeds [4,13,31]. Prior to the pull-off measurement, the rough surface to be tested was first attached to the microscope stage. The fibre array with the acrylic backing was placed on the surface to obtain initial self-alignment. Then, a small droplet of glue (Loctite Instant Adhesive 495, Loctite) was applied to the back of the acrylic peg using a needle point. The stem of the load cell was then brought down until it lightly contacted the glue. The glue was then let dry for 20 min. The fibre array being glued to the load cell stem when in alignment with the rough surface facilitated repeatable subsequent pull-off experiments between well-aligned surfaces. In addition, the alignment and contact between the samples was observed through the optical microscope enabled by the translucent acrylic substrate. The need to check the alignment and contact limited this study to the use of only acrylic substrates as rough probes.

In the pull-off experiments, the fibre array was lowered at 10 μm s^−1^ speed to contact the adhering surface and reach a compressive load of 700 mN, the load after which the fibres comprising the array were observed to buckle. After this compressive load was reached, surfaces were kept in contact for 10 s followed by the retraction of the fibre array at 10 μm s^−1^ until detachment. Since the automated stages are feedback controlled, they maintain constant speed throughout each experiment, which allows us to estimate distance travelled with time. Both time and forces were recorded during each experiment to generate the force–distance curves. A given fibre array was tested 10 times on each rough surface in descending order of the surface RMS roughness, from the roughest to the smoothest. A number of individual fibres from the FG-4 µm and FG-7 µm samples were observed to rupture during the very first adhesion experiment on the smoothest, the 54 nm surface, potentially due to the large tensile stresses fibres experience. Thus, the first two test results for the functionally graded samples are excluded from the averages and standard deviations in the results section. The FG-7 µm contacting the 54 nm surface and fibre rupture at the end of the test is shown in electronic supplementary material, movie S1. The movie was recorded during the second test and the sample includes ruptured fibres from the previous run in addition to the fibres ruptured during the current test.

## 3. Results

**Figure 3.**
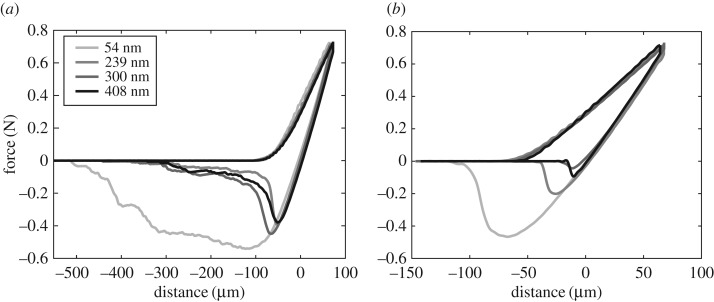
Sample force–distance data obtained from (*a*) FG-7 µm and (*b*) monolithic fibre arrays on all the adhering surfaces.

Shown in figure 3*a,b* are the sample force–distance data obtained with the monolithic and FG-7 µm on all testing surfaces, respectively. Since we cannot identify when the first contact between the sample and the surface occurs, we assumed the zero displacement to be the zero load point in the retract phase of the experiment. The first adhesion metric of interest to be calculated from the force–distance data is the pull-off stress, which is a measure of adhesion strength. It is an average value and is calculated by dividing the pull-off force, which is the magnitude of the minimum force in the force–distance curve, by the area of the adhesive sample. The area of the samples is 2.5 mm × 2.5 mm and constant among all fibre arrays. Visual inspection of the force–distance data suggests that FG-7 µm sample is affected significantly less from substrate roughness used in this study in terms of pull-off stress compared with the monolithic sample.

**Figure 4.**
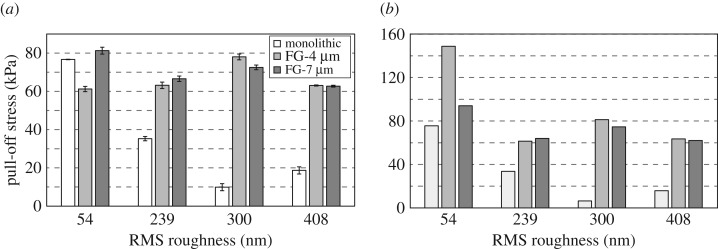
(*a*) Average pull-off stress for FG-4 µm, FG-7 µm and monolithic fibre array on all the adhering surfaces. (*b*) Pull-off stress measured for the very first test prior to potential fibre rupture.

Average pull-off stresses for all samples are shown in figure 4*a*. While the pull-off stress of the monolithic sample drops dramatically from 77 kPa on the 54 nm surface to 19 kPa on the 408 nm surface, that of the FG-4 µm sample drops from 81 kPa only to 63 kPa on the same adhering surfaces. Data indicates that pull-off stress of the FG-7 μm is not influenced adversely by roughness included in this study, even showing an increase with intermediate roughness. However, these deductions are not entirely accurate for the functionally graded samples because of the fibre rupture issue observed after the very first test on the smoothest 54 nm surface as mentioned in the previous section. Since measurements were performed on the rougher surfaces first and no fibre rupture was observed on these surfaces, results were not affected by fibre rupture on surfaces rougher than the 54 nm surface. A more realistic picture of the effect of roughness on pull-off stress can be observed in figure 4*b*, which includes the pull-off stress obtained from the very first experiment on each adhering substrate. Since no fibre rupture is observed, figure 4*a*,*b* seem similar for the monolithic sample, showing 75% less pull-off stress on the roughest surface (19 kPa) compared with the smoothest one (77 kPa). Both functionally graded samples adhere significantly better to the smoothest testing surface in the first test than in the subsequent ones due to fibres breaking during the first test. Referring to figure 4*b*, the FG-4 µm sample loses about 57% pull-off stress going from the smoothest to the roughest surface (149–64 kPa), whereas the FG-7 µm surface loses only about 34% (94–62 kPa) on the same adhering surfaces. Both the FG-4 µm and the FG-7 µm exhibit pull-off stresses more than threefold that of the monolithic sample on the 408 nm surface.

**Figure 5.**
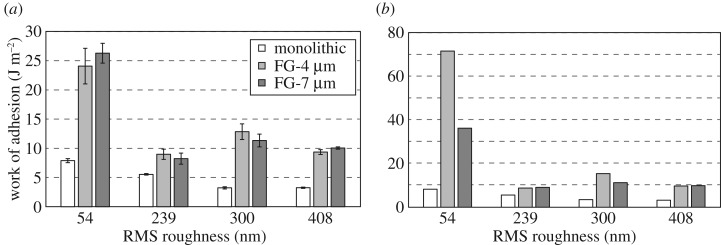
(*a*) Average effective work of adhesion for the FG-4 µm, FG-7 µm and monolithic fibre array on all the adhering surfaces. (*b*) Effective work of adhesion measured for the very first test prior to potential fibre rupture.

Figure 5*a*,*b* show the average effective work of adhesion and the effective work of adhesion from the very first measurement on each surface, respectively. The effective work of adhesion is calculated by dividing the adhesion hysteresis (the area between the approach and retract curves) by the area of the adhesive sample. We will again look at the results from the very first measurement in figure 5*b*, similar to the pull-off stress results, in order to isolate the effect of roughness on the effective work of adhesion. Here, the influence of roughness is more detrimental to the effective work of adhesion than to the pull-off stress. This time, the monolithic sample maintains the effective work of adhesion relatively well and loses 61% from the smoothest to the roughest surface (8.3 J m^−2^ to 3.2 J m^−2^). The functionally graded sample with the thicker distal layer, FG-7 μm, lost 73% (36.4 J m^−2^ to 9.9 J m^−2^) compared with 86% (71.9 J m^−2^ to 9.7 J m^−2^) for the FG-4 μm sample when tested on the same smooth (54 nm RMS) and rough (408 nm RMS) surface. These values are on the order of three orders of magnitude larger than typical work of adhesion values observed for most elastomers. This can be attributed to the contribution of the elastic energy stored in the fibres prior to detachment to the adhesion hysteresis [34].

**Figure 6.**
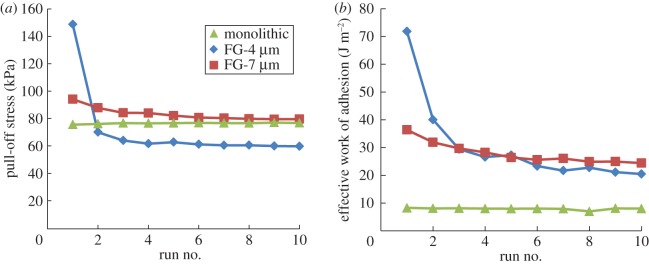
(*a*) Pull-off stress and (*b*) the effective work of adhesion as a function of the test run on the 54 nm RMS surface.

Figure 6 shows the effect of fibre rupture and the lack thereof for the functionally graded samples and the monolithic sample, respectively. This data is obtained on the smoothest adhering surface with 54 nm RMS roughness, the only adhering surface which led to fibre rupture. The pull-off stress and the effective work of adhesion dropped 60% and 72% after 10 measurements for the FG-4 μm sample, respectively. The FG-7 μm sample followed it by 15% drop in pull-off stress and 33% in effective work of adhesion. The performance of the monolithic sample showed no change with the number of tests as no fibre rupture was observed during repeated tests.

## 4. Discussions

**Figure 7.**
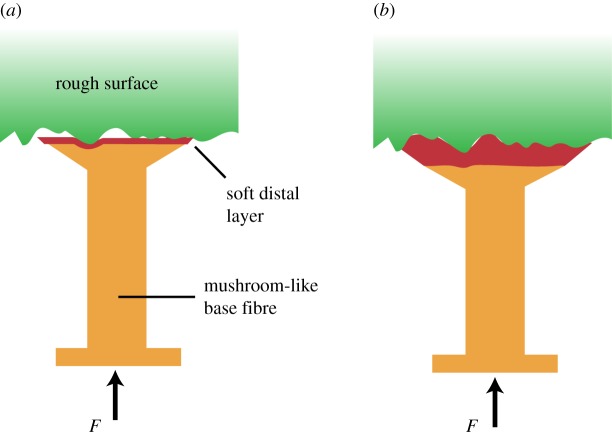
An illustration of the contact of an individual fibre with a given rough surface for a functionally graded fibre with (*a*) thin and (*b*) thick soft distal layer. For a given compressive load *F*, the fibre with the thicker distal layer is expected to create a larger contact area with the adhering surface due to higher compliance.

The increase in contact compliance due to the softer distal layer has competing consequences for rough surface adhesion. As shown by Minsky & Turner [35], where they looked at the effect of the thickness of a soft layer on a stiff core, the pull-off force increased with decreasing thickness of the soft layer. A similar effect was observed by Balijepalli *et al.* [36] when they performed experiments with a 2 mm diameter stiffer stalk (*E* = 2 MPa and *E* = 350 MPa) covered with a softer distal layer (*E* = 0.9 MPa). Both their numerical analysis and experiments showed that smooth surface adherence increases with decreasing thickness of the soft distal layer. Owing to similar confinement effects, Aksak *et al.* has also shown numerically and experimentally that pull-off stress increases with decreasing aspect ratio for soft elastomer posts [37]. This is because a thinner film acts stiffer due to confinement effects and favours a more uniform stress distribution at the contact interface. Our experimental results on the smoothest surface support these findings in that the FG-4 μm sample has a higher pull-off stress than the FG-7 μm sample, even though the fibres comprising the FG-7 μm sample are on average approximately 15% larger. The thickness of the soft distal layer has implications on the local compliance and contact area as well. As depicted in figure 7, for a given compressive load, a fibre with a thicker distal layer will generate larger contact area because the deformation will be mostly confined in the softer distal layer. And because the distal layer is very soft, the penalty in the stored elastic energy will be low, increasing the effective work of adhesion as shown in equation (1.1). Thus, for functionally graded fibres, while a thicker distal layer compromises adhesion strength to smoother surfaces, the added compliance will minimize the effect of roughness on pull-off. As the layer gets thinner, the limited compliance due to the confinement effect will hurt rough surface adhesion but enhance the smooth surface pull-off stress. These two competing factors that depend on the thickness of the distal layer, namely the uniformity of the stress distribution and compliance, pose an optimization problem and together determine the adhesion strength for a given surface. Our experimental results support these hypotheses. In a recent study, which is an extension of [36], Fischer *et al.* [38] looked at the effect of distal layer thickness on pull-off force when the composite fibres were tested on both a smooth (average peak-to-valley roughness *R*_z_ = 41 nm) and a rough surface (*R*_z_ = 2.174 µm). They found that pull-off force increased by three to five times compared with conventional monolithic pillar structures made out of the soft distal layer material. Moreover, specifically for pillars with a curved interface between the stalk and the soft distal layer, the pull-off force increased with decreasing distal layer thickness on both the smooth and rough surfaces, and the dependence of pull-off force to distal layer thickness was very similar for both surfaces. This is due to the larger size scale of the tested pillar, 20 µm was the thinnest soft layer included in their testing. This thickness is much larger than the 2.174 µm peak-to-valley roughness of their rough surface and thus the rough surface results closely resemble the smooth surface results in their study. On the contrary, the layer thicknesses used in our work are closer to the roughness values of the tested surfaces and thus the thickness of the distal layer has a more prominent effect on pull-off force as shown in experimental results.

Surprisingly, our study shows that the roughness has a more detrimental effect on the effective work of adhesion than on the pull-off stress for the functionally graded samples in particular. Referring to figure 3*b*, the tensile load decreases very gradually after the pull-off load is reached for the 54 nm adhering surface but the reduction in the tensile load is more abrupt for the remaining rougher surfaces. This indicates that more fibres stay attached to the 54 nm surface after the pull-off load is reached, which suggest a higher level of stochasticity and explains the dramatically larger effective work of adhesion. The displacement when the pull-off is reached is not influenced heavily by roughness, pointing to a less stochastic behaviour when functionally graded fibres are in contact with rougher surfaces.

The type of functional grading in material properties as shown in this work also exists in nature. Ladybird beetles were recently shown to have a gradient in the material composition and elastic modulus along the individual fibres of their attachment pads, a negative gradient in elastic modulus moving from the proximal to the distal end [39]. This finding suggests that some of the natural fibrillar adhesives use a similar strategy as presented here to reduce the dependency of adhesion to roughness amplitude.

The observations in this study bring about another issue with bioinspired adhesives that is often overlooked. Fibres that adhere strongly also experience very large stresses. In our previous study, we showed that a fibre can experience stresses as high as 8.8 MPa, which is not only much higher than the elastic modulus of the fibre but is also higher than the reported tensile stress for the polyurethane used in that study [31]. Note that even the use of polyurethane, a material known for its high tear and tensile strength, cannot prevent rupture for highly adhesive fibres. This puts a limit to the attainable adhesion for fibre arrays made of soft elastomers. However, functional grading could solve this problem by enabling the use of stiffer materials for fibre construction. The lack of contact compliance can be remedied with a softer distal layer without compromising the durability and the strength provided by a stiffer material used for the base fibre. Additionally, enabling the use of stiffer materials would lead to larger fill factors and higher aspect ratios without fibre collapse, potentially enhancing adhesion strength even further.

Despite promising results, the sub-micron roughnesses used in this work are much lower than the tens of millimetres that the gecko can cope with on a regular basis [40]. Note that gecko's seta diameter is an order of magnitude smaller than that of the fibres employed in this work while their height is similar [1]. Achieving similar dimensions to gecko seta using synthetic fibres with similar fibre fill factors is only possible if we were to use materials of elastic modulus similar to beta-keratin, which is of the order of 1–4 GPa. However, this material lacks the necessary local compliance to stick to rough adhering surfaces. Using functionally graded materials can solve this problem by providing sufficient local compliance for adhesion and still achieve very high fill factors using a stiff material for the fibre stalk. The overall adhesion will then be determined by the ability of the fibres to bend, stretch and buckle to conform to the global roughness. Future efforts will, therefore, focus on shrinking fibre diameters and studying adhesion to rougher adhering surfaces.

## 5. Conclusion

Functionally graded fibre arrays have been proposed as rough surface adhesives in this work. Unlike monolithic fibre arrays whose adhesive strength drops dramatically with increasing roughness, functionally graded fibre arrays showed strong adhesion to both smooth and rough testing surfaces, exhibiting much lower dependency on the roughness values featured in this work. Specifically, the functionally graded fibres with a thicker distal array performed equally well on both smooth and rough testing surfaces. Fibres with the thinner distal layer performed significantly better on the smoothest testing surface, despite being affected more adversely with increasing surface roughness. Both functionally graded samples showed more than a threefold improvement over the monolithic sample when tested on the roughest adhering surface. The effective work of adhesion decreased at a much higher rate than the pull-off stress for both the monolithic and the functionally graded samples with increasing surface roughness. Fibre rupture was determined to be an issue for the functionally graded fibres specifically on smooth surfaces. As a result, pull-off stress dropped rapidly with each measurement for the functionally graded fibre arrays showing as high as 60% decrease after 10 pull-off experiments. Our results suggest that functionally graded fibres have the potential to mimic natural fibrillar adhesives in their ability to strongly attach to both rough and smooth surfaces and show promise as universal repeatable adhesives. This potential can be realized if in fact functionally graded fibre adhesives were to strongly adhere to surfaces with RMS roughness amplitudes of tens of millimetres. Thus, we will investigate the coupled relationship between the thickness of the distal layer, the diameter of the fibre, the ratio of elastic moduli of the materials comprising the fibres and the topography of the adhering surface as they relate to adhesion and seek to optimize these parameters for peak pull-off stress. In awareness of the elastic moduli and the size of the attachment elements found in nature, we expect fibres with smaller diameters in the range 2–10 µm, high fill factors, and high aspect ratios to achieve strong adhesion to rough surfaces.

## Supplementary Material

Power spectral density data of the tested surfaces

## Data accessibility

All data used in this research are included in figures, table and the electronic supplementary material. The experimental movie and the power spectral density plots of the adhering surfaces are also available in the electronic supplementary material.
