# Supplementary figures and images for: Sticking to rough surfaces using functionally graded bio-inspired microfibres
Source: R Soc Open Sci. 2017 Jun 7;4(6):161105. doi: 10.1098/rsos.161105 (PMC5493905; doi:10.1098/rsos.161105)

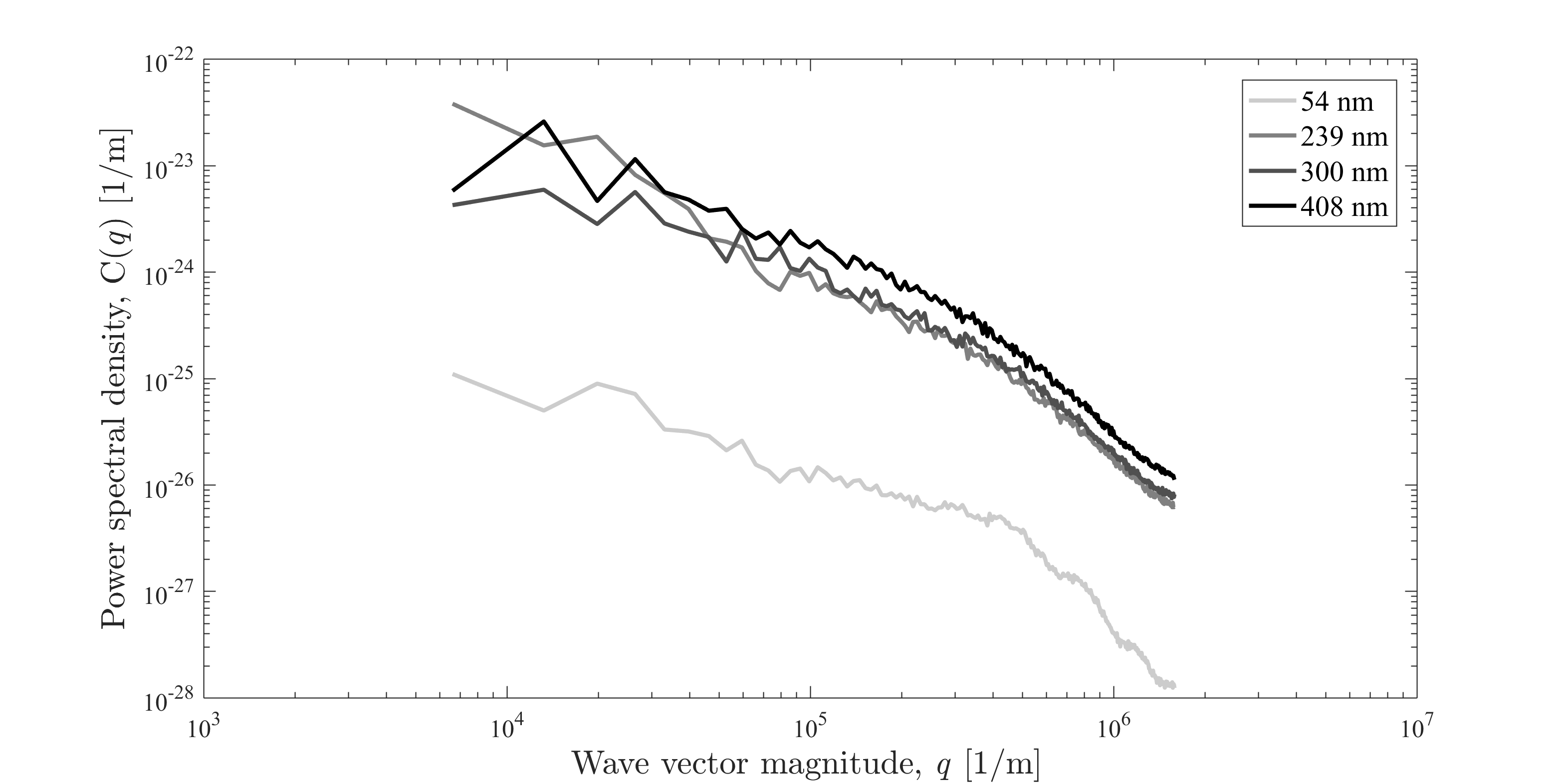

Supplement: Power spectral density data of the tested surfaces [file rsos161105supp1.png]
